# Epistatic and Functional Interactions of Catechol-O-Methyltransferase (COMT) and AKT1 on Neuregulin1-ErbB Signaling in Cell Models

**DOI:** 10.1371/journal.pone.0010789

**Published:** 2010-05-24

**Authors:** Yoshitatsu Sei, Zhen Li, Jian Song, Renee Ren-Patterson, Elizabeth M. Tunbridge, Yukihiko Iizuka, Masahiro Inoue, Berenice T. Alfonso, Senda Beltaifa, Yoko Nakai, Bhaskar S. Kolachana, Jingshan Chen, Daniel R. Weinberger

**Affiliations:** 1 Clinical Brain Disorder Branch, Genes, Cognition, and Psychosis Program, National Institute of Mental Health, National Institutes of Health, Bethesda, Maryland, United States of America; 2 Department of Psychiatry, Warneford Hospital, University of Oxford, Oxford, United Kingdom; 3 Department of Infectious Diseases, Kurume University of Medicine, Kurume, Fukuoka, Japan; University Medical Center Groningen, Netherlands

## Abstract

**Background:**

Neuregulin1 (NRG1)-ErbB signaling has been implicated in the pathogenesis of cancer and schizophrenia. We have previously reported that NRG1-stimulated migration of B lymphoblasts is PI3K-AKT1dependent and impaired in patients with schizophrenia and significantly linked to the catechol-o-methyltransferase (COMT) Val108/158Met functional polymorphism.

**Methodology/Principal Findings:**

We have now examined AKT1 activation in NRG1-stimulated B lymphoblasts and other cell models and explored a functional relationship between COMT and AKT1. NRG1-induced AKT1 phosphorylation was significantly diminished in Val carriers compared to Met carriers in both normal subjects and in patients. Further, there was a significant epistatic interaction between a putatively functional coding SNP in AKT1 (*rs1130233*) and COMT Val108/158Met genotype on AKT1 phosphorylation. NRG1 induced translocation of AKT1 to the plasma membrane also was impaired in Val carriers, while PIP_3_ levels were not decreased. Interestingly, the level of COMT enzyme activity was inversely correlated with the cells' ability to synthesize phosphatidylserine (PS), a factor that attracts the pleckstrin homology domain (PHD) of AKT1 to the cell membrane. Transfection of SH-SY5Y cells with a COMT Val construct increased COMT activity and significantly decreased PS levels as well as NRG1-induced AKT1 phosphorylation and migration. Administration of S-adenosylmethionine (SAM) rescued all of these deficits. These data suggest that AKT1 function is influenced by COMT enzyme activity through competition with PS synthesis for SAM, which in turn dictates AKT1-dependent cellular responses to NRG1-mediated signaling.

**Conclusion/Significance:**

Our findings implicate genetic and functional interactions between COMT and AKT1 and may provide novel insights into pathogenesis of schizophrenia and other ErbB-associated human diseases such as cancer.

## Introduction

Neuregulin1 (NRG1)-ErbB signaling has been implicated in the pathogenesis of cancer and schizophrenia. In recent work using a B lymphoblast cell model, we showed that these cells express a functional ErbB signaling pathway, and that NRG1 promotes their adhesion and migration [1], [2]. Using this cell system, we found that NRG1-stimulated cell adhesion and migration were impaired in patients with schizophrenia [1], [2]. Further, we demonstrated that the COMT Val158Met polymorphism, which alters COMT enzyme activity [3], [4], [5], predicts the adhesive and migratory responses of these cells to NRG1 in both normal subjects and in patients and that the group of patients with the high-activity Val allele was the most relatively impaired. Because NRG1-mediated adhesion and migration is PI3K-AKT1dependent [1], [2], the findings from our previous studies led us to hypothesize that there may be a functional relationship between COMT and AKT1. Interestingly, both COMT and AKT1 have also been implicated in the pathogenesis of cancer and schizophrenia, and their associations with these disease conditions have been extensively studied [4], [6].

COMT catalyzes the transfer of the methyl group of S-adenosylmethionine (SAM) to a hydroxyl group on a variety of catechols, including the catecholamine neurotransmitters and carcinogenic catechol estrogen compounds [4]. A G to A transition (*rs4680*) at COMT codon 108/158 translates a valine to methionine substitution (Val158Met) that codes for high and low activity proteins, respectively [3], [4]. A number of studies have shown that the high activity Val allele is associated with relatively poorer function of prefrontal cortex, compared with the Met form [7], [8], [9], [10], [11]. While results have been inconsistent, the Val allele has also been occasionally implicated as a risk factor for schizophrenia [7], [12]. Similarly, some studies have also shown an association of Val108/158Met with breast cancer [13], [14], [15], [16], [17], [18] and prostate cancer [19]; the low activity Met allele is often associated with increased risk of metastasis and poorer prognosis. Recent evidence indicates that there are other functional variants in COMT that modulate the biologic effects of the Val/Met polymorphism and confound simple Val/Met associations [20], [21].

AKT1 is a serine/threonine kinase that functions as a central element in the cell survival pathway and is activated in many cancers [22]. Recently, a Glu to Lys mutation at amino acid 17 of the AKT1coding sequence has been identified in breast, colon and ovarian cancers, suggesting that this mutation may cause 2-8% of cancers of these types [23]. The AKT1 gene has also been associated with schizophrenia. Emamian and colleagues [24] demonstrated reduced AKT1 protein expression in B lymphoblasts and postmortem brain tissues from patients with schizophrenia. They also provided the first evidence of association between AKT1 and schizophrenia in Caucasians based on 5 SNPs spanning this gene. At least six subsequent studies [24], [25], [26], [27], [28] and one-meta analysis [29] have provided supportive evidence for this association. Further, a coding and risk-associated polymorphism (*rs1130233*) has been associated with low expression of the protein and increased susceptibility to apoptosis in B lymphoblasts [30], [31]. AKT1 also has been implicated in dopamine signaling in brain [32], [33], and a recent imaging study of normal subjects suggested that COMT and AKT1 interact at the level of cortical physiology related to dopamine function [31].

Despite the overlap of COMT and AKT1 in associations with cancer and schizophrenia, molecular and cell biological interactions of COMT and AKT1 have not been explored. In the present study, using NRG1-ErbB signaling in the B lymphoblast cell system and in other cell models, we show evidence of epistatic effects between these genes in that COMT Val/Met affects AKT1 translocation and activation by competing for methyl donor availability required for PS synthesis. Our findings provide a potential mechanism for the impact of COMT on NRG1-induced adhesion and migration and also possibly for the association of these genes with schizophrenia and cancer.

## Results

### Effects of the COMT Val/Met polymorphism on NRG1-stimulated phosphorylation of AKT1

**Figure 1:**
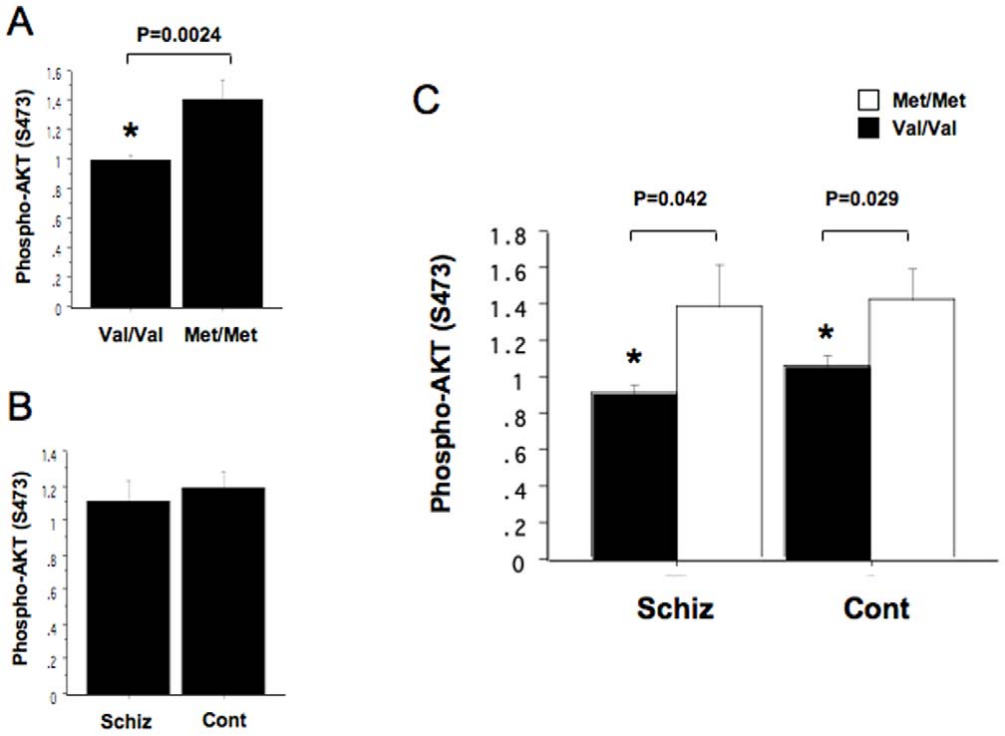
Effects of COMT Val/Met genotype and disease on NRG1-stimulated Ser-473 phosphorylation of AKT1in B lymphoblasts derived from controls and patients with schizophrenia. (A) COMT Val/Meet genotype and NRG1-stimulated Ser-473 phosphorylation of AKT1. NRG1-stumulated increases in AKT1 phosphorylation in B lymphoblasts were significantly decreased in Val homozygotes (n = 18), compared with Met homozygotes (n = 12) (p = 0.0024, t-test). The Y-axis shows the peak fold increases in the phosphorylated-AKT1/total AKT1 ratio during one hour stimulation. (B) Disease and and NRG1-stimulated phosphorylation of AKT1. There was no difference in NRG1-stumulated increases in AKT1 phosphorylation in B lymphoblasts between controls (n = 16) and patients with schizophrenia (n = 14). (C) COMT Val homozygotes had significantly lower NRG1-stimulated phosphorylation of AKT1 in both controls (P = 0.029) and patients with schizophrenia (p = 0.042, t-test).

We previously demonstrated an association between COMT Val/Met (*rs4680*) genotype and NRG1-mediated adhesion and migration [1], [2]. Because NRG1-mediated adhesion and migration are PI3K/AKT1 signaling-dependent, we sought to determine if NRG1-induced activation of AKT1 was also associated with COMT genotype. Activation of AKT1 depends on its phosphorylation at Thr-308 in the catalytic domain and Ser-473 in the C terminus. In B lymphoblasts, NRG1 significantly induced a Ser-473 phosphorylation of AKT1 that persisted for at least 1 hour, whereas total AKT1 levels remained unchanged. Therefore, we measured peak fold increases in the Ser-473 phophorylated-AKT1/total AKT1 ratio over baseline during one hour of NRG1-stimulation. Using this assay, we found a significant effect of COMT genotype on NRG1-stimulated Ser-473 phosphorylation of AKT1 (Figure 1). Thus, consistent with our earlier preliminary findings [1], [2], NRG1-stumulated increases in AKT1phosphorylation were significantly decreased in Val homozygotes, compared with Met homozygotes (p = 0.0024) and this was unaffected by diagnosis (Figure 1).

### Epistatic effects of COMT Val/Met and AKT1 rs1130233 on NRG1-stimulated phosphorylation of AKT1

**Figure 2:**
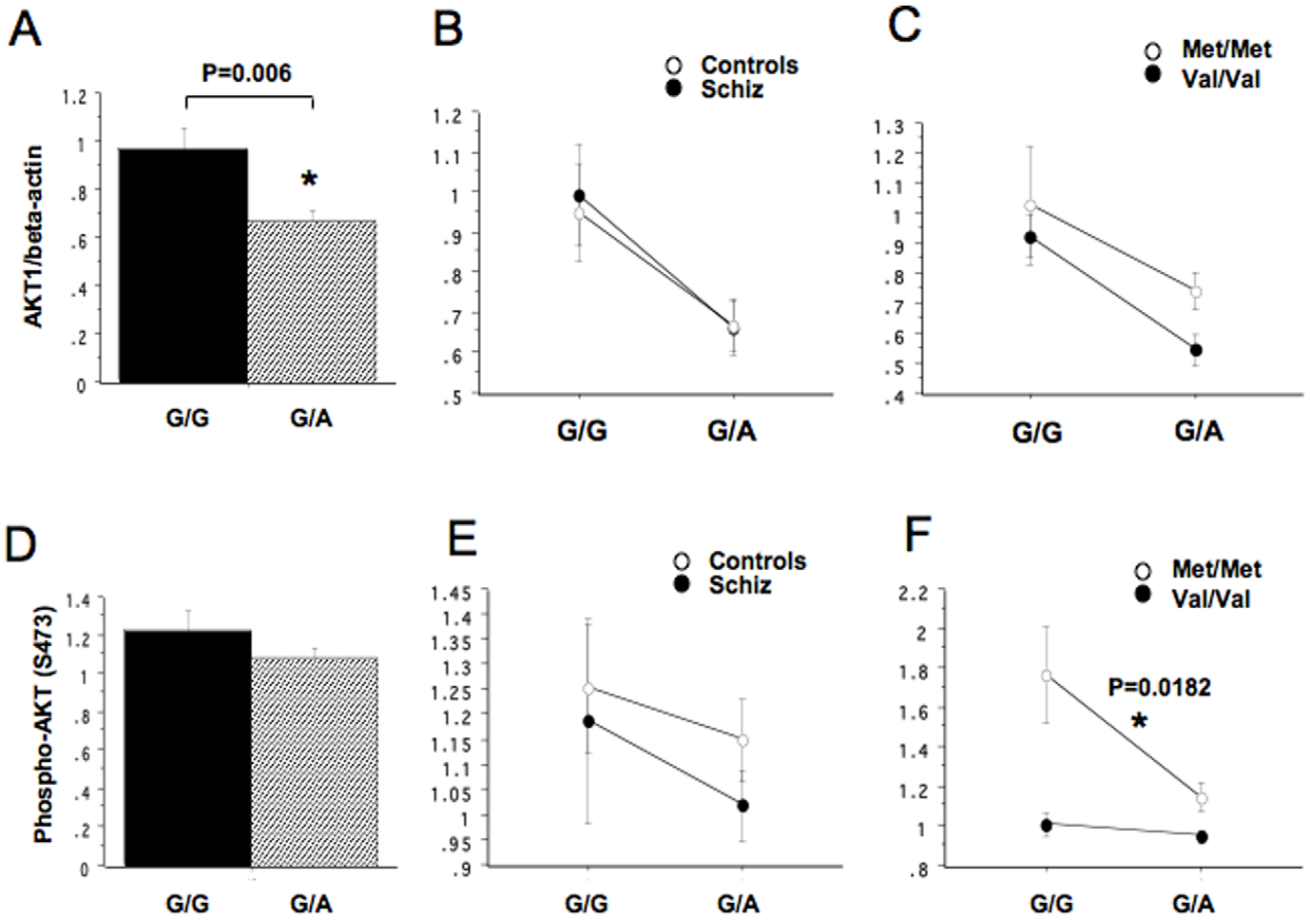
Effects of AKT1 *rs1130233* on AKT1 protein expression and NRG1-stimulated Ser-473 phosphorylation of AKT1in B lymphoblasts derived from controls and patients with schizophrenia. (Upper panel; A, B and C) Effects of AKT1 *rs1130233* on AKT1 protein expression. The minor *rs1130233* allele was associated with lower expression of AKT1 protein (AKT1/β-actin ratio, mean±SE). p = 0.006, *rs1130233* G/A vs. G/G; p = 0.0406. The associations were preserved in both control and patient groups (B), similarly in COMT Val/Val and Met/Met groups (C). Within the *rs1130233* G/A group (C), AKT1protein expression was significantly decreased in individuals with Val carriers compared with Met carriers. p = 0.0371, *rs1130233* G/A, Val vs. G/A, Met. The number of subjects carrying [G/G, Val], [G/G, Met], [G/A, Val] and [G/A, Met] genotype was 18, 12, 10 and 16, respectively. (Lower panel; D, E and F) Effects of AKT1 *rs1130233* on NRG1-stimulated phosphorylation of AKT1. AKT1 *rs1130233* had no effect on this phenotype in the total population (D) and no interaction with disease on the phenotype (E). Two-way ANOVA showed significant main effects of AKT *rs1130233* genotype (P  = 0.0066), significant main effect of COMT genotype (p = 0.0003) and significant interactions of these genotypes (p = 0.0234) on NRG1-induced phosphorylation. In individuals who were also COMT Met/Met homozygotes, individuals carrying the AKT1 S *rs1130233* minor A allele, showed significantly lower phosphorylation than G/G carriers (P = 0.0182). This effect was not appeared in Val/Val individuals because of the maximum reductions in AKT1 phosphorylation.

To confirm whether a functional polymorphism within the AKT1 gene is associated with the expression and function of AKT1 and might interact epistatically with COMT, we genotyped 64 B lymphoblast lines for the coding SNP *rs1130233* previously found to affect AKT1 protein levels and radiation induced apoptosis [30]. AKT1 *rs1130233* genotype was in Hardy-Weinberg equilibrium (Fisher's exact test p-values >0.05) and showed a minor allele frequency (MAF) similar to that reported previously for Caucasian populations (Supplementary Information, Fig. S1 online). Consistent with earlier studies [24], [30], [31], we also confirmed that levels of AKT1 protein, assessed by Western blot and displayed as AKT1/β-actin ratios, were significantly lower in heterozygotes (G/A) compared with G/G homozygotes (p = 0.006) (Figure 2).

In contrast to the association with protein levels, *rs1130233* genotype showed no association with NRG1-stimulated phosphorylation of AKT1 in the entire sample (Figure 2D) and no interaction with disease status (Figure 2E), but it did interact epistatically with COMT Val/Met genotype (Figure 2F). A two-way ANOVA revealed a significant interaction between AKT1 *rs1130233* genotype and COMT Val/Met genotype (F(1, 25) = 5.83, p = 0.0234) on NRG1-induced Ser-473 phosphorylation of AKT1(Figure 2F). Post-hoc tests showed that the interaction was due to a significant *rs1130233* genotype effect on NRG1-induced Ser-473 phosphorylation of AKT1 only in individuals who were COMT Met/Met homozygotes (P = 0.0182), likely because Val/Val individuals had markedly reduced phosphorylation of AKT1 regardless of *rs1130233* genotype. We found no interaction for AKT1 protein levels (Figure 2C).

### Effect of increasing COMT activity on AKT1 phosphorylation

**Figure 3:**
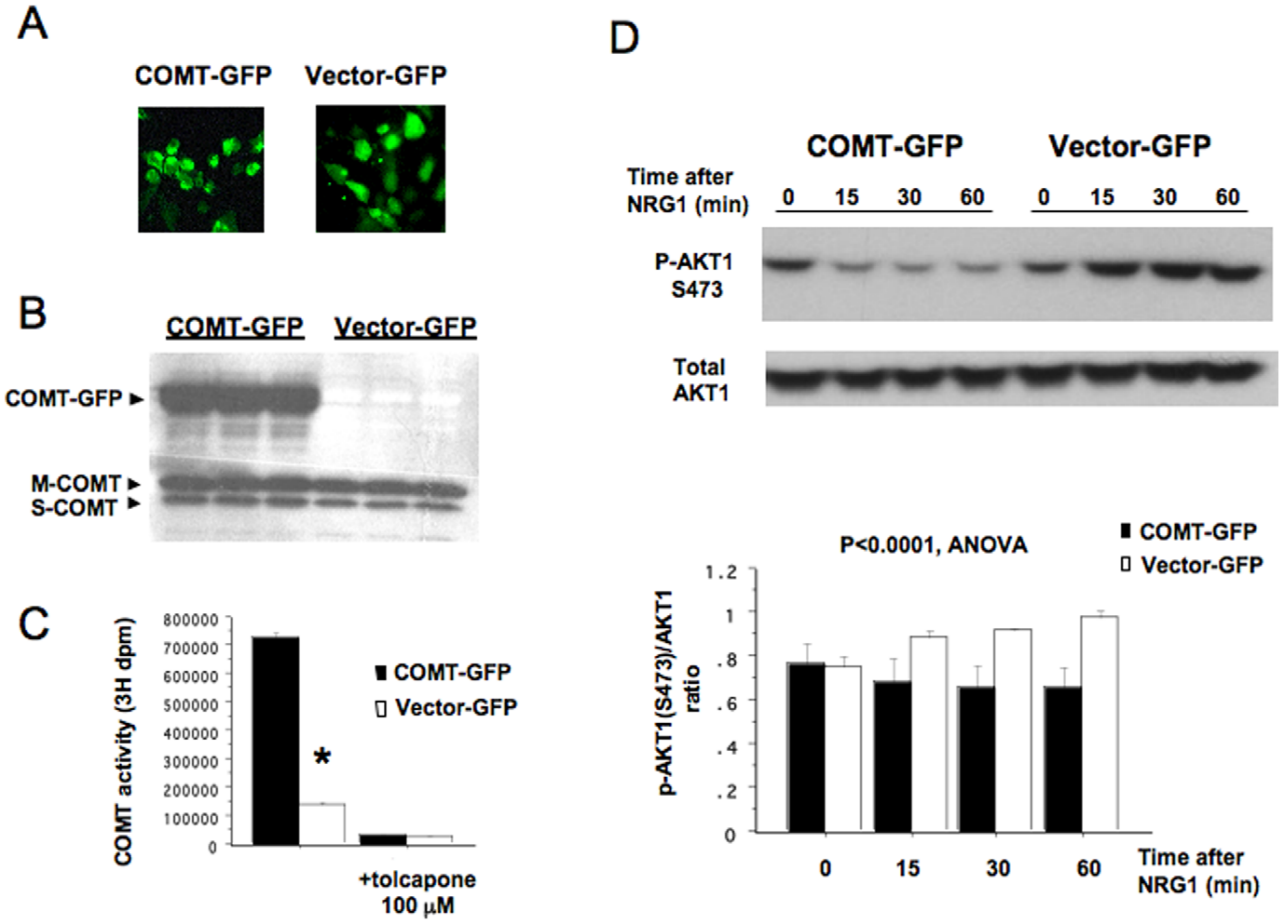
Effects of COMT transfection on COMT activity and NRG1-stimulated Ser-473 phosphorylation of AKT1 in SH-SY5Y cells. SH-SY5Y cells were transfected with COMT-GFP or with the control GFP vector. After 48 hrs, cells were analyzed for enzyme activity and expression of COMT and tested for Ser-473 phosphorylation of AKT1 in response to NRG1α. (A) GFP-detection after transfection of GFP-tagged COMT or control GFP vector showed 70–80% transfection efficiency. (B) Expression of the transfected COMT. Western blotting showed expression of a 60-kDa band, representing GFP-tagged human MB-COMT in cells transfected with COMT-GFP (left three lanes). Endogenous 30-kDa MB- and 25-kDa S- COMT proteins were expressed under both transfection conditions. (C) Effects of COMT transfection on COMT enzyme activities. The COMT enzyme assay indicated a significant five-fold increase in COMT activity (dpm per mg total protein) in cells transfected with COMT-GFP compared to cells transfected with control vector. *p<0.0001, 3 samples per group. The specificity of the assay was confirmed by a complete attenuation of the enzyme activity by 100 µM of the specific COMT inhibitor tolcapone. (D) Effects of COMT transfection on NRG1α-stimulated Ser-473 phosphorylation of AKT1 in SH-SY5Y cells. At 48 hrs after transfection, the transfected cells were stimulated with NRG1α (100 ng/ml) for the time points indicated. Protein isolated from the cells was analyzed by Western blotting. To quantify the level of phosphorylation, the immunoblots were stained with antibodies specific to phosphorylated AKT1- at Ser-473 and were then stripped and reprobed with antibodies to total AKT1. The bar graphs represent changes in the ratio of phosphorylated form to total AKT1 (means±SEM, from 3 transfection experiments). A repeated measure ANOVA showed a significant main effect of COMT transfection on AKT1 phosphorylation (P = 0.0017).

The COMT Val/Met polymorphism is associated with variable enzyme activity: the Val allele encodes an enzyme with higher activity than the Met form [3], [5]. Therefore, we hypothesized that decreases in NRG1-stimulated AKT1 phosphorylation in COMT Val homozygote lymphoblasts were due to high COMT activity. To determine the effect of increasing activity of COMT on AKT1 phosphorylation, we overexpressed the Val form of COMT in SH-SY5Y cells. The overexpressed COMT transcript was tagged with GFP, allowing us to monitor transfection efficiency by measuring GFP positive cells using fluorescence microscopy (Figure 3A). We maintained consistently high transfection efficiency, which was between 70% and 80%. In these transfected cells, we confirmed expression of GFP-tagged COMT protein as well as endogenous membrane-bound (MB) and soluble (S) forms of COMT protein by Western blot (Figure 3B). We found a five-fold increase in COMT activity in these cells, compared with those transfected with control vector, and demonstrated the specificity of the enzyme activity assay by showing that measurement of COMT activity was almost completely blocked by the addition of the specific COMT inhibitor tolcapone (Figure 3C).

SH-SY5Y cells express ErbB2, 3 and 4 receptors and activate typical tyrosine receptor signaling cascades in response to NRG1-stimulation, such as a PIP_3_-AKT1 signaling cascade (unpublished observations). Consistent with these observations, NRG1 increased AKT1phosphorylation that persisted for at least 60 min in SH-SY5Y cells transfected with a control vector containing GFP only. In contrast, NRG1-stimulated phosphorylation of AKT1 was significantly reduced in COMT transfected cells (Figure 3D). Repeated measures ANOVA showed a significant main effect of COMT transfection on AKT1phosphorylation (F3,12 = 19.91, p = 0.0017) (Figure 3D).

### COMT Val/Met genotype and translocation of PHD-AKT1 in B lymphoblasts

**Figure 4:**
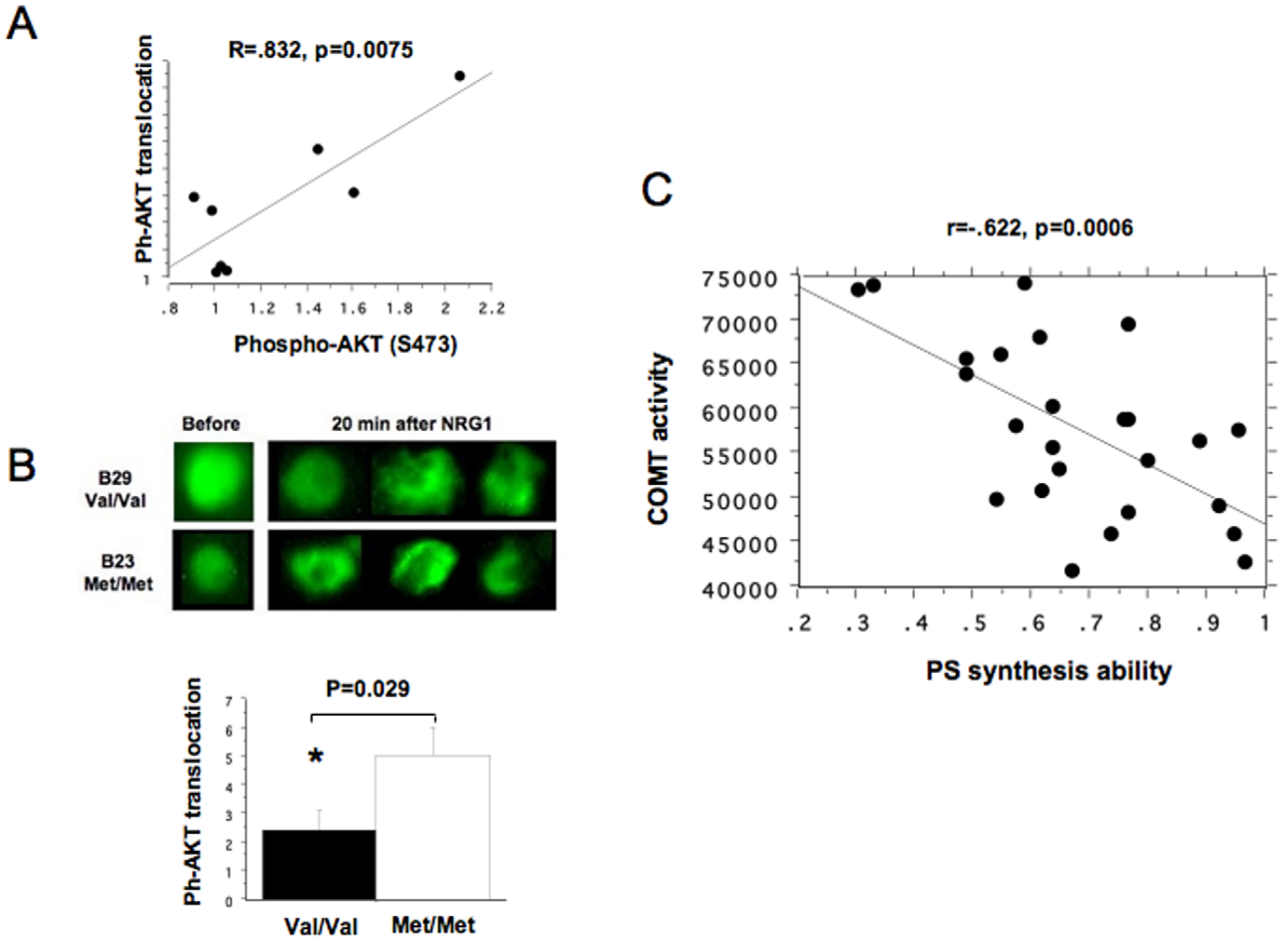
Effects of COMT Val/Met genotype on PHD-AKT1translocation and phospholipid levels in B lymphoblasts derived from controls and patients with schizophrenia. (A) There was a positive correlation between NRG1-stimulated Ser-473 phosphorylation of AKT1 and NRG1-simulated PHD-AKT translocation. B lymphoblasts from control subjects were transfected with pEYFP-ph-AKT. At 24 hrs after transfection, PHD-AKT1 translocation was stimulated with NRG1 for 15 min and measured under the fluorescence microscope. Translocation indexes from 8 control subjects were positively correlated with NRG1-stimulated peak fold increases in Ser-473 phosphorylation of AKT1 (phophorylated-AKT1/total AKT1 ratio) that were separately obtained from the same individuals (r = .832, p = 0.0075). (B) Representative fluorescence microscopic images of PHD-AKT1 fluorescence. Single cell images of PHD-AKT1fluorescence before and after NRG1 stimulation in typical COMT Val/Val and Met/Met homozygotes are shown. The bar graph represents PHD-AKT1 tranlocation index (means±SEM). *p = 0.029, individuals with Val/Val genotype (n = 5) vs. Met/Met genotype (n = 5). (C) There was a correlation between COMT enzyme activity and phosphatidylserine (PS) synthesis ability. Y-axis indicates relative COMT activity (dpm per mg total protein) in the B lymphoblasts. X-axis indicates PS synthesis ability (the ratio of total PS fluorescence intensity of B lymphoblast after exposure to serum free medium for 24 hrs/PS fluorescence intensity after culturing in normal culture media containing 15% FBS, which reflects a cell's ability to maintain PS levels) (see Materials and Methods). COMT enzyme activity was significantly correlated with PS synthesis ability in total 25 subjects (12 patients and 13 controls) (Total; R = −.622, p = 0.0006, patients; R = −.667, p = 0.0156 and controls; R = −.634, p = 0.0180).

PIP_3_-triggered translocation of AKT1 from the cytoplasm to the plasma membrane is a prerequisite for its phosphorylation and activation [34]. To examine this process in cells, we constructed a vector containing fluorescence–tagged PHD-AKT1 (pEYFP-ph-AKT) and developed a method to evaluate translocation using B lymphoblasts transfected with this construct. B lymphoblasts from 8 control individuals were used for this test (Figures 4A and B). PHD-AKT1 translocation was examined using fluorescence microscopy 24 hours after transfection, before and after stimulation with NRG1. Firstly, we found a significant positive correlation between NRG1-stimulated translocation and NRG1-stimulated Ser-473 phosphorylation of AKT1 (phosphorylated-AKT1/total AKT1ratio)(r = .832, p = 0.0075) (Figure 4A), consistent with the notion that NRG1-stimulated phosphorylation depends on the translocation of AKT1. Secondly, we found that NRG1-stimulated translocation was significantly lower in Val homozygotes than in Met homozygotes (p = 0.029) (Figure 4B). These results suggest that the difference in NRG1-stimulated Ser-473 phosphorylation between Val and Met homozygotes is due to differences in AKT1translocation.

#### COMT, phosphatidylserine and PIP_3_ in B lymphoblasts

Although PS has no direct effect on AKT1 activity [35], it has been implicated as playing a role in PIP3-induced full AKT1 activation since the translocation of AKT1 may require the interaction of PHD-AKT1with membrane PIP_3_ and PS [36]. For this reason, we studied the status of PIP_3_ and PS in the B lymphoblasts. PS levels in B lymphoblasts from 25 subjects were measured after 16 hr of serum-deprivation and compared with those from cells fed normally. The serum deprivation, which removes exogenous PS, PC and cholines, reduced PS levels (paired t-test, mean diff; −12.015, p<0.0001). However, the degree of the reduction varied widely depending on the subject. Thus, the ratio of PS levels in serum-deprived condition/PS level in the non-deprived condition, a measure of PS synthetic capacity, from 25 subjects ranged between 0.3 and 1.0. These ratios were inversely correlated with COMT enzyme activity measured in the same lymphoblast lines (r = −.622, p = 0.0006) (Figure 4C). The higher the COMT activity cells possess, the greater the accompanying reduction in PS synthetic capacity. This result suggests a functional relationship between COMT activity and the cells' ability to synthesize PS.

Another potential mechanism for the poor NRG1-stimulated phosphorylation of AKT1associated wtih COMT Val genotype or high enzyme activity might be reduced PIP_3_ generation. However, NRG1-stimulated PIP_3_ generation was not diminished (Supplementary Information, Fig. S2 online). Wald-Wolfowitz Runs Test indicated no significant effect of COMT Val/Met genotype on NRG1-stimulated PIP_3_ production (p = 0.711), and there was no effect of AKT1 rs1130233 genotype (P = 0.6488) and no interaction.

### Effects of high COMT activity on NRG1-stimulated translocation of PHD-AKT1 and PIP_3_ generation in SH-SY5Y cells

**Figure 5:**
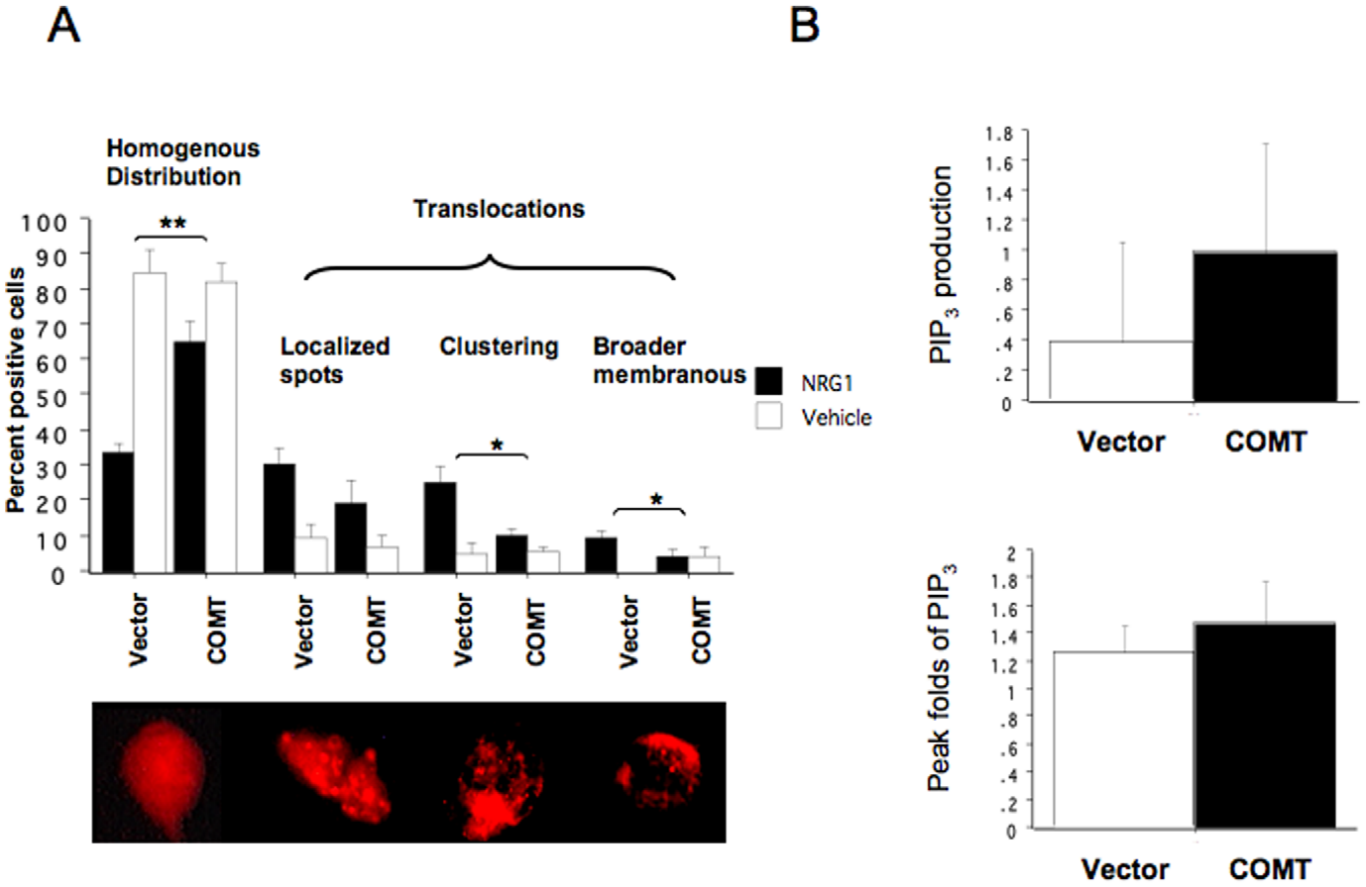
Effects of high COMT activity on NRG1-stimulated PHD-AKT1 translocation and PIP_3_ generation in transfected SH-SY5Y cells. (A) Effects of high COMT activity on PHD-AKT1translocation. SH-SY5Y cells were double-transfected with either pDsRed-ph-AKT plus COMT or pDsRed-ph-AKT plus control vector. After 48 hrs, cells were analyzed for PHD-AKT1 translocation in response to NRG1α. The bar graph represents percent positive cells indicating PHD-AKT1 translocation (means±S.E.) from four transfection experiments. Two-way ANOVA, interaction between NRG1α treatment and COMT transfection, *p<0.05, **p = 0.0074. Lower inset shows representative fluorescence microscopic images of PHD-AKT1 fluorescence in a single cell. From left, untranslocated homogenous distribution of PHD-AKT1, translocated PHD-AKT1 as spots, clusters and broad membranous distributions. (B) Effects of high COMT activity on NRG1-stimulated PIP_3_ generation. SY5Y cells were transfected with either COMT or control vector. The cells were analyzed for NRG1-stimulated PIP_3_ generation using flow cytometry. Top panel indicates a summation of positive and negative changes in PIP_3_ during a 30 min observation period and lower panel indicates peak folds of PIP_3_ during a 30 min period.

To further determine if effects of COMT Val/Met genotype on NRG1-stimulated translocation of PHD-AKT1 in B lymphoblasts is due to COMT enzyme activity, we performed an AKT1 translocation experiment using the COMT-transfected SH-SY5Y cell model. pDsRed-ph-AKT was used for this experiment since COMT was tagged with GFP. Our examinations of fluorescence signals confirmed that there was no overlapping signal from green (GFP) and red (pDsRed) fluorescence, indicating that the localization of red fluorescence reflected accurately the localization of PHD-AKT1 expressed in the transfected cells. Approximately 48 hours after double-transfection with either pDsRed-ph-AKT plus pAcGFP-N1-COMT or pDsRed-ph-AKT plus control vector, the cells were stimulated with NRG1 and terminated by fixation buffer at different time points. This time-course study indicated that stimulation with NRG1 produced PHD-AKT1 localization, which was observed as fluorescence distribution patterns of multiple spots, clusters or broad membranous distribution (Figure 5A). The results from three independent experiments showed that the proportion of cells with homogenous distribution (i.e., no translocation) was significantly lowered after NRG1 treatment in the cells transfected with control vector compared to the COMT transfected cells, suggesting that NRG1-stimulated translocation of PHD-AKT1 in COMT-transfected cells was significantly suppressed compared to the cells transfected with control-vector. A two-way ANOVA showed a significant interaction between NRG1-treatment and COMT-transfection for the NRG1-induced changes in proportion of cells with homogenous distribution (i.e., no translocation) (F(1,12) = 10.34, p = 0.0074) (Figure 5A). The NRG1-induced increases in the cells showing clusters and broad membranous distribution of PHD-AKT1 were significantly different between COMT and control vector-transfected cells. There were significant interactions between NRG1-treatment and COMT-transfection for those two categories (F(1, 12) = 5,44, p = 0.0379 for clustering and F(1,12) = 5.31, p = 0.0398 for membranous distribution) (Figure 5A). These results from the PHD-AKT1 translocation experiments suggested that significant reductions in NRG1-stimulated Ser-473 phosphorylation in the COMT-transfected cells was due at least in part to poor AKT1 translocation.

We then studied NRG1-stimulated PIP_3_ generation to determine if the poor NRG1-stimulated translocation and phosphorylation of AKT1 by the COMT transfection is due to reduced PIP_3_ generation. However, there was no difference in NRG1-stimulated PIP_3_ generation between the COMT- and control vector-transfected cells in two measures from three independent transfection experiments: summation of changes (P = 0.5782) and peak folds (p = 0.6134) (Figrure 5B). These results from the SH-SY5Y transfection system were consistent with those from B lymphoblasts.

### Effects of COMT on AKT1 activation and PS levels are reversed by SAM

**Figure 6:**
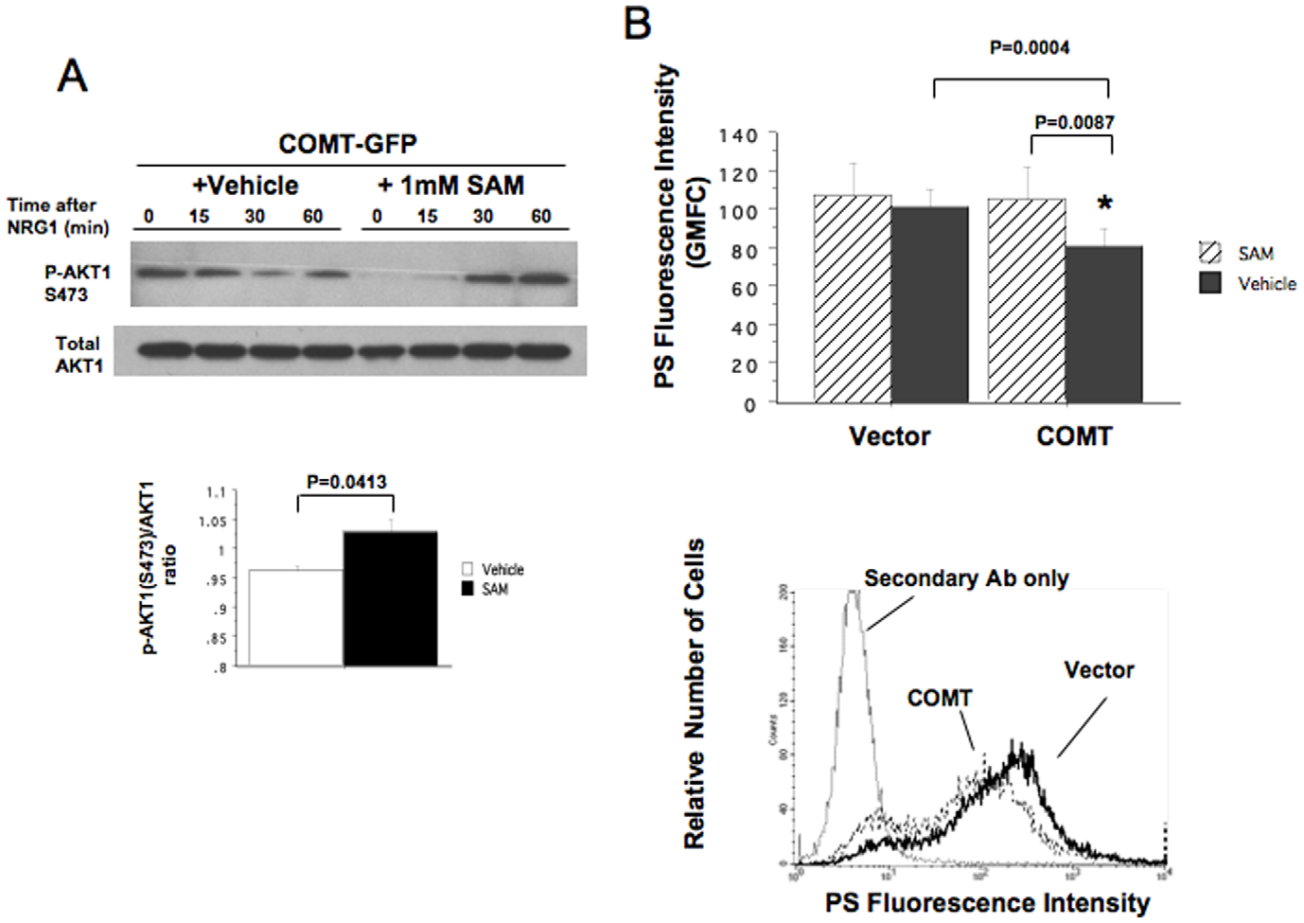
Effects of SAM on NRG1-stimulated AKT1 phosphorylation and PS amount in transfected SH-SY5Y cells. (A) Effects of SAM treatment on NRG1-stimulated Ser-473 phosphorylation of AKT1 in SH-SY5Y cells overexpressing COMT. At 48 hrs after transfection with COMT-GFP, cells were treated with either 1 mM SAM or vehicle for 60 minutes and stimulated with NRG1α (100 ng/ml). The immunoblot shows a representative time course of Ser-473 phosphorylation of AKT1and expression of total AKT1 after NRG1α. The bar graph represents the ratio of phosphorylated- to total AKT1 at 60 min after the stimulation (means±SEM, from 3 transfection experiments). p = 0.0413, vehicle vs. SAM treatment. (B) Effects of COMT transfection on total PS amount in SH-SY5Ycells. SH-SY5Y cells were transfected with COMT-GFP or with control GFP vector. After 48 hrs, cells were and treated with either 1 mM SAM or vehicle control for 60 min and analyzed to estimate total PS amount of cells by flow cytometry. A graph indicates PS fluorescence intensity (geometric mean) from two independent transfection experiments. A repeated measure of ANOVA showed significantly lower PS fluorescence intensity in COMT-transfected cells than that in cells transfected with control vector (*P = 0.0087) and a significant reversal of the transfection effect by SAM treatment (*p = 0.0004).

PS synthesis is regulated by constitutively active methylation of phospholipids [37]. The enzymes PEMT, PSS1 and PSS2 are responsible for maintaining the balance of PE, PC and PS, particularly when PC and PE are in limited exogenous supply [37]. We hypothesized that the reductions in AKT1phosphorylation and PS synthesis caused by COMT transfection may be mediated by a disruption of phospholipid methylation. This is a plausible mechanism, since PEMT uses the same methyl donor (S-adenosylmethionine; SAM) as COMT. Therefore, COMT activity might indirectly impact on the function of PEMT (and vice versa) due to competition for SAM. If this is the case, the effect of COMT on AKT1phosphorylation and on PS synthesis should be reversible by SAM supplementation. Indeed, in the COMT-transfected SH-SY5Y cells, SAM treatment reversed the inhibitory effect of COMT transfection on NRG1-stimulated phosphorylation of AKT1 (Figure 6A), supporting this hypothesis. The ratio of phosphorylated/total AKT1 at 60 min after the stimulation was significantly increased by 1 mM SAM treatment prior to the stimulation (p = 0.0413, vehicle vs. SAM). Using the SH-SY5Y cells, we also found that COMT transfection decreased total PS levels significantly; ANOVA revealed a significant effect of COMT transfection (F(1,7) = 38.6, P = 0.0004). Further, SAM treatment significantly reversed the COMT transfection effect on PS (F(1,10) = 10.55, p = 0.0087) while there was no interaction between COMT transfection and SAM treatment (Figures 6B). These results are consistent with the inverse relationship between COMT activity and PS synthesis ability seen in B lymphoblasts (Figure 4C) and also suggest that the increase in COMT activity reduces PS synthesis and NRG1-stimulated phosphorylation of AKT1 in a SAM-dependent manner.

Because the neuroblastoma line SH-SY5Y is dopaminergic and these cells express dopamine receptors, it is conceivable that the effect of COMT transfection on PS might be mediated by dopamine in these cells. Therefore, we also tested HEK293 cells, which normally do not express dopamine receptors [38]. Consistent with our data obtained in SH-SY5Y cells, we found that COMT transfection decreased PS to a greater extent than control vector transfection (p = 0.0191, unpaired t-test) (Supplementary Information, Fig. S3 online) and this COMT-induced reduction in PS was reversed by SAM treatment (Supplementary Information, Fig. S4 online). In this experiment, we also tested whether active removal of SAH by SAHH transfection could have either an additive or synergistic effect with SAM treatment, since SAH acts as a functional inhibitor of SAM-dependent methyltransferases. However, we found no significant effect of SAHH transfection alone or an interaction between SAHH transfection with SAM treatment on PS synthesis, suggesting that the effect of COMT on PS synthesis is mediated by insufficient SAM levels, rather than excessive SAH accumulation.

### Effects of COMT on AKT1 activation are not restricted to NRG1-ErbB signaling

**Figure 7:**
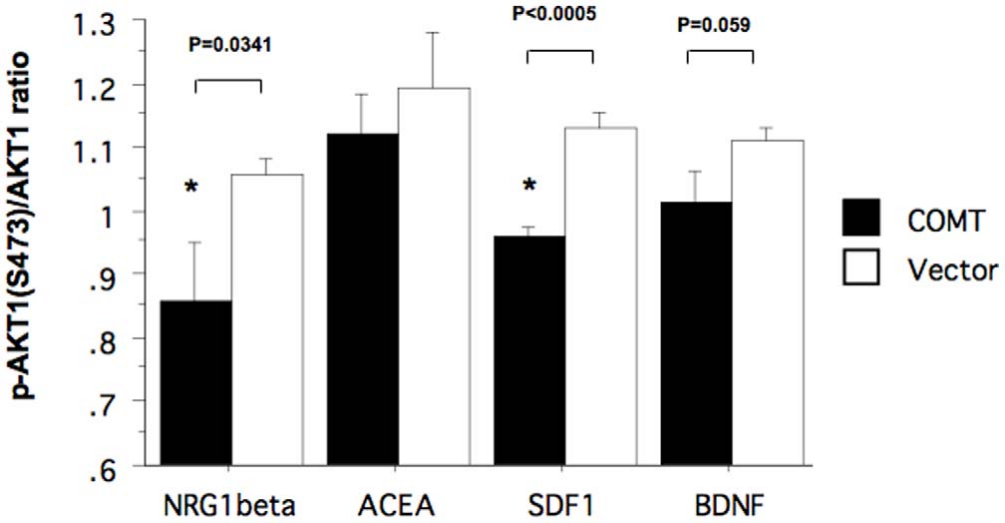
Effects of COMT transfection on Ser-473 phosphorylation of AKT1 after stimulation with various ligands in transfected SH-SY5Y cells. At 48 hrs after transfection, the transfected SH-SY5Y cells were stimulated with NRG1β (100 ng/ml), cannabinoid CB1 agonist ACEA (14 nM), SDF1β (50 ng/ml) or BDNF (50 ng/ml). The bar graphs represent maximum changes in the ratio of phosphorylated- to total AKT1 during the 60-minutes observation period (means±SEM, from 3–4 transfection experiments per ligand).

If the decrease in PS synthesis is the cause, at least in part, for the poor translocation and phosphorylation of AKT1, the effect of COMT Val/Met genotype or enzyme activity may not be limited to NRG1-ErbB signaling. To test this hypothesis, we studied whether COMT transfection affects ligand-stimulated phosphorylation of AKT1 induced via other signaling pathways, using SH-SY5Y cells. We used BDNF to stimulate the tyrosine kinase receptor trkB, and SDF1 and ACEA to stimulate the G-protein coupled receptors, CXCR4 and the cannabinoid (CB) receptor, respectively. We also tested the β-isoform of NRG1 to confirm that the effect of COMT transfection on NRG1-ErbB-mediated phosphorylation of AKT1is not specific to the α-isoform. Although the inhibition in ACEA or BDNF-stimulated AKT1phosphorylation by COMT overexpression was small, COMT over-expression significantly inhibited SDF1-stimulated phosphorylation of AKT1 (p<0.0005, t-test) (Figure 7). Furthermore, consistent with our findings for NRG1α, we confirmed significant suppression of AKT1phosphorylation following stimulation with NRG1β (P = 0.0341) (Figure 7). These results suggest that COMT enzyme activity, which is related to Val/Met genotype, affects AKT1activity stimulated by a variety of ligands, and that the mechanism(s) for this is dopamine-independent.

### Effects of high COMT activity on NRG1-induced migration of SH-SY5Y cells

**Figure 8:**
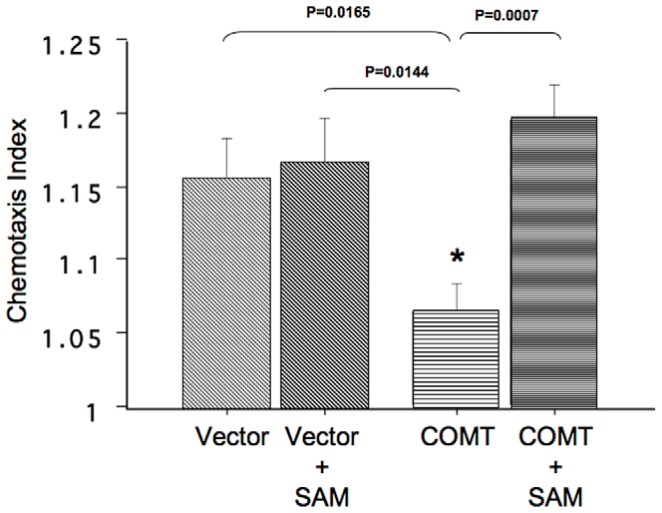
Effects of COMT transfection and SAM treatment on NRG1-stimulated migrataion in SH-SY5Y cells. SH-SY5Y cells were transfected with COMT-GFP or control vectors (empty GFP) and cultured in media containing 2%FBS. After 48 hrs, cells were tested for NRG1-stimulated migration. A graph indicates the chemotaxis index (mean± S.E.) from three transfection experiments (n = 4–5 per group). ANOVA showed a significant a SAM treatment effect (p = 0.0193) and transfection-treatment interaction (P = 0.0144).

Finally, we attempted to determine if high COMT enzyme activity produces a negative effect on cell migration. Since SH-SY5Y cells also migrate in response to NRG1 in a PI3K/AKT1-dependent manner, the SH-SY5Y-COMT transfection system is suited for this experiment. As expected in untransfected SH-SY5Y cells, our migration assay using a transwell chamber showed a positive NRG1-stimulated migration in control-vector transfected cells (Figure 8). In contrast, COMT transfection significantly decreased NRG1-stimulated migration compared to the transfection with a control empty vector (p = 0.0165, unpaired t-test) (Figure 8). Further, SAM treatment significantly rescued the COMT transfection effect on migration (Figure 8). Repeated measures ANOVA revealed a significant main effect of SAM treatment (F1, 12 = 7.30, P = 0.0193) and a significant interaction between COMT transfection and SAM treatment (F1, 12 = 8.18, p = 0.0144). These results are consistent with the effect of COMT Val/Met genotype on NRG1-stimulated migration seen in B lymphoblasts [2] and therefore suggest that the increase in COMT activity reduces migration ability in a SAM-dependent manner.

## Discussion

In the present study, we have found that the valine allele of COMT is associated with diminished NRG1-induced AKT1 phosphorylation in B lymphoblasts from both controls and patients, and showed that COMT overexpression in SH-SY5Y cells led to impaired AKT1 phosphorylation and migration in response to NRG1. These results suggest that the relatively poorer NRG1-induced adhesion and migratory response seen in Val homozygote lymphoblasts [2] is due, at least in part, to reduced activation of AKT1. In addition, we have demonstrated a plausible mechanism by which the effect of COMT activity on AKT1 function may be mediated, at least in part. We suggest that consumption of SAM by COMT may affect the ability of cells to regulate PS levels involved in translocation and activation of AKT1 by altering phospholipid methylation, although clearly we cannot rule out the possibility that the competition of COMT for SAM impacts on other potential mechanisms (e.g. DNA methylation) that could affect AKT1 phosphorylation in addition to changes in PS or independent of such changes and could, moreover, be the more important mechanism of the effect of COMT on AKT1 phosphorylation.

As with other chemokine-stimulated migratory responses, activation of AKT1is essential for NRG1-stimulated cell adhesion and migration in B lymphoblasts, evidenced by the severe attenuation of adhesion and migration by PI3K or AKT1 inhibition in these cells [1], [2]. Therefore, we suggest that the poor migratory response we previously showed in Val homozygote lymphoblasts might be due at least in part to poor activation of AKT1 in these cells. It is likely that the association between the COMT Val/Met polymorphism and AKT1 activation is mediated via its effects on COMT enzyme activity [3], since COMT overexpression in SH-SY5Y cells significantly decreased NRG1-induced phosphorylation of AKT1. Taken together, these data suggest that high COMT activity (resulting either from overexpression or as the result of Val homozygosity) is inhibitory to the function of AKT1.

Identifying the mechanism of how COMT activity inhibits the function of AKT1, and therefore, NRG-dependent adhesion and migration is challenging. Considering the well characterized function of COMT as an enzyme involved in catechol inactivation, it was conceivable that the effect of COMT on migration and adhesion could be related to a dopamine- or catecholic estrogen-mediated mechanism, since these substrates for COMT can be derived from fetal bovine serum or might be produced by B lymphoblasts in culture. However, this mechanism is unlikely to be responsible, since in B lymphoblasts the production of dopamine is low and the expression of dopamine receptors is scarce (unpublished observation), and because the migration assay was performed in serum-free media. Rather, our earlier results suggested another mechanism, an indirect effect of COMT on methylation of other critical molecules involved in phospholipids to explain the inverse relationship between COMT and AKT1 activation.

It has been suggested that an increase in COMT-mediated methylation decreases the SAM pool and increases SAH, which acts as a feedback inhibitor of SAM-dependent methylation processes [4]. Because the Val form of COMT has higher enzyme activity, it would result in relatively higher SAM consumption and SAH generation than the Met form, a hypothesis supported by data showing higher levels of plasma homocysteine, a molecule formed by the hydrolysis of SAH, in Val carriers compared with Met homozygotes [39]. Therefore, Val homozygotes would be predicted to have a greater inhibitory effect of COMT on other SAM-dependent methyltransferases, compared with Met homozygotes. Among these methyltransferases, we considered phosphatidylethanolamine *N*-methyltransferase (PEMT) to be a good candidate for mediating COMT's association with AKT1 function, as it is involved in the synthesis of PS, which is important for the full activation of AKT1. Activation of AKT1 requires its translocation from the cytoplasm to the plasma membrane, followed by its phosphorylation at residue T308 and then S473 by PDK1 and PDK2, respectively. It has been well demonstrated in many systems that translocation of AKT1 is triggered by PIP_3_ levels, which are regulated by PI3K and PTEN [40]. However, its translocation has been found to not only require membrane PIP3, and but also PS [36]. PS serves as an essential attractor for AKT1 by providing a negative charge to the PHD-AKT1. Consistent with this theory, translocation of AKT1 has been shown to be affected dose-dependently by PS content [41].

After confirming that PEMT, PSS1 and PSS2, which mediate the synthesis of PS [37], are expressed in B lymphoblasts (Supplementary Information, Fig. S5 online), we predicted that high COMT activity might decrease PS in cells via competition with PEMT for SAM, especially in serum-free culture, where exogenous PS, PC and cholines are unavailable. Consistent with this prediction, we found that overnight culture in serum-free media caused significant decreases in PS content in B lymphoblasts and that the extent of the decrease was negatively correlated with COMT activity. Thus, the higher COMT activity the cells possessed, the bigger decreases in PS the cells exhibited. We also showed that NRG1-stimulated translocation of PHD-AKT1 is significantly lower in Val homozygotes than in Met homozygotes while NRG1-stimulated production of PIP_3_ was not reduced in Val homozygotes compared to Met homozygotes. Therefore, these results were consistent with the hypothesis that COMT's effects on AKT1 are mediated via PS, rather than PIP_3_. The impaired NRG1-stimulated PHD-AKT translocation without altering PIP3 generation was observed in COMT-transfected SH-SY5Y cells. Moreover, the inverse relationship between COMT and PS was also supported by COMT transfection studies using SH-SY5Y cells. The transfection studies also demonstrated that the deficit in AKT1 activation induced by COMT overexpression could be rescued by SAM administration, demonstrating the SAM-dependence of COMT's negative regulation of AKT1 function. Thus, our results suggest that high COMT activity affects the cells' ability to maintain or synthesize PS via the PEMT-PSS1 pathway, leading to reduced PS content and, in turn, poorer translocation and activation of AKT1.

If the reduction in PS synthesis and AKT1 activation is related to COMT-mediated SAM consumption, we would expect that these effects of COMT on AKT1 would not be limited only to NRG1-ErbB signaling. Consistent with this prediction, studies using SH-SY5Y and HEK293 cells demonstrated that COMT overexpression inhibited phosphorylation of AKT1 in other relevant signaling pathways such as SDF1-CXCR4, and similar to our findings for NRG1, this inhibition was overcome by SAM augmentation.

Interestingly, in addition to a single effect of COMT genotype on AKT1, we also found evidence of an interaction between COMT Val/Met and a SNP in AKT1 that is associated with variation in AKT1 protein expression. Thus, the minor A allele of *rs1130233*, which is associated with lower AKT1 protein levels in lymphoblasts, was associated with poorer NRG1-induced AKT1 phosphorylation in cells that also were in COMT Met homozygotes. This statistical interaction is not based on a simple differential effect of COMT Met and Val alleles on AKT1 phosphorylation in the context of AKT1 genotype, since Val alleles produced maximum inhibition of NRG1-induced AKT1 phosphorylation regardless of AKT1 genotype. Because AKT1 protein levels would be expected to have an effect on AKT1 recruitment and localization for its activation and because of the association of *rs1130233* genotype with AKT1protein levels [30], we would expect the effect of the SNP in AKT1 to be more apparent in Met COMT carriers. Consistent with prior data, we were able to replicate the association between AKT1 *rs1130233* and protein levels [30], demonstrating that the minor A allele of *rs1130233* is associated with low levels of AKT1 protein but not with changes in AKT1 transcript expression (Supplementary Information, Fig. S6 online). Thus we suggest that the low levels of protein in the carriers of the *rs1130233* A allele are likely caused by a post-translational modification, i.e., degradation and turnover rate of the protein, perhaps related to its association with scaffold proteins such as 14-3-3 and HSP90 etc [40]. According to the finding by Harris et al [30], it seems likely that AKT1 *rs1130233* itself is not a functional SNP, since its association with AKT1 protein levels is not found in African Americans, who show very low LD in this genomic region. Therefore they suggested that another relatively common SNP or combination of SNPs, causes the post-translational reduction of the protein. However, our replication of Harris et al's report confirm that *rs1130233* can serve as a surrogate marker for protein levels in populations with high LD in this region, such as Caucasians.

Since COMT, AKT1, and ErbB-signaling are each implicated in both cancer and schizophrenia, NRG1-ErbB signaling in B lymphoblasts provides a biologically plausible research tool for elucidating cellular mechanisms relevant to both cancer biology and neurobiology. Using this system, we demonstrated epistatic effects of COMT Val/Met and AKT1 *rs1130233* on AKT1 activation. The effects were confirmed and some of the potential mechanisms explored using COMT transfection of cell lines. Effects of COMT Val/Met in cancer biology have almost always been discussed in the context of its role in detoxifying carcinogens and estrogens. Similarly, in neurobiology, the COMT Val/Met polymorphism has most often been linked with cortical dopamine metabolism. The present findings offer an additional facet to COMT's biology that has new, broad and potentially important implications for cancer biology and schizophrenia via its interaction with AKT1 (Supplementary Information, Fig. S7 online). AKT1 signaling mediates a number of critical neuronal signaling molecular pathways that are implicated in schizophrenia and also plays a central role in tumorigenesis in many cancers. If the potential for an AKT1x COMT gene-gene interaction is taken into consideration in future association studies, we would predict that the minor A allele of AKT1 *rs1130233* might strengthen associations between COMT Val/Met and the multiple phenotypes with which it has been associated, including cognitive function, schizophrenia and cancer. Consistent with this prediction, a recent report highlighted epistatic interactions of COMT Val/Met and AKT1 *rs1130233* on cortical physiology assayed with neuroimaging and on cognitive functions [31]. Thus, future studies of epistatic interactions between genes such as COMT and AKT1 might help in elucidating the relationship between schizophrenia and cancer that has been discussed in epidemiological studies [42].

## Materials and Methods

### Subjects

The B lymphoblast cell lines used for this study are derived from the same subjects described in our previous reports [1], [2]. The subjects are age- and sex-matched and all Caucasians of self-reported European ancestry to avoid genetic stratification and to reduce heterogeneity. Importantly, all subjects were selected and matched on the basis of homozygosity at the catechol-o-methyltransferase (COMT) Val158Met locus to increase statistical power to study the biological effects of the COMT Val/Met polymorphism. These subjects were drawn from individuals participating in the Clinical Brain Disorders Branch “Sibling Study” protocol, an ongoing investigation of neurobiological abnormalities related to genetic risk for schizophrenia. The details of subject recruitment and examination are described previously [43]. Blood collection and transformation of lymphocytes were approved by the NIMH institutional review board, and all donors provided written informed consent. The number of samples included in each assay varied based on the availability of cells at the time of testing.

### Genotype determination

Genotypes at *rs1130233* (G>A) in AKT1 and *rs4680* in COMT (G>A, val108/158met) were determined by 5′ exonuclease allelic discrimination TaqMan assay using probes and primers available from ABI as part of their “Assays on Demand” program.

### Cell cultures and stimulations

B lymphocytes in a mononuclear cell preparation from the subjects were transformed by infection with EBV and maintained as previously described [44]. SH-SY5Y and HEK293 cells were purchased from the American Tissue Culture Collection (ATCC, Manassas, VA). The transformed B lymphoblasts, SH-SY5Y and HEK293 cells were grown in RPMI-1640 medium (Gibco, Grand Island, NY) containing 15% heat-inactivated fetal bovine serum (FBS) (Cambrex, Walkersville, MD), L-glutamine (2 mmol/L), 100 µg/mL streptomycin and 100 units/mL penicillin (Gibco) in an incubator (95% air/5% CO_2_ at 37°C). For stimulation experiments, cells were stimulated with 100 ng/ml neuregulin1α (296–HR) (R & D system, Inc, Minneapolis, MN), which is a 65 amino acid residue recombinant protein from the EGF domain of NRG1α (aa177–241) For some experiments, cells were stimulated with 100 ng/ml NRG1β, 50 ng/ml BDNF, 50 ng/ml SDF1β, 50 ng/ml FGFb, or 14 nM arachidonyl-2′-chloroethylamide (ACEA). NRG1β (377-HB), BDNF (248-BD) and SDF1β (351-FS) were purchased from R & D system, Inc. FGFb (F0291) was from Sigma-Aldrich Inc, St. Louis, MO. The highly selective CB1 receptor agonist ACEA (1319) was from Tocris (Ellisville, MO).

### Immunoblot

Immunoblot and analysis were performed as previously described [2]. Antibodies to AKT1 (07–416) and phospho-AKT1 (S473) (05–669) were purchased from Upstate (Charlottesville, VA). An antibody to phospho-AKT1 (S473) (9271) was from Cell Signaling Technology (Danvers, MA). Anti-phospho-AKT1 (T308) antibody (ab4796) was from Abcam (Cambridge, MA). Anti-β-actin monoclonal antibody (A5441) was from Sigma (St. Louis, MO). To detect the primary antibodies, horseradish peroxidase-conjugated anti-rabbit or mouse IgG antibody (Pierce Biotechnology, Rockford, IL) was used.

### Flow cytometric analysis of cellular phosphatidylserine levels and PIP3 levels

Relative PS levels at the single cell level were assessed by flow cytometry after staining both extracellular and intrallular PS with anti-PS antibody (4B6) (Abcam Inc., Cambridge, MA) using the Cytofix/Cytoperm kit (BD Biosciences, San Jose, CA). Relative PIP_3_ levels were assessed using anti-PIP_3_ antibody (Echelon Biosciences Inc., Salt Lake City, UT). Briefly, cells were fixed with Phosflow Fix Buffer I (BD Bioscience) for 10 min at 37°C. Cells were then washed with Phosflow Perm/Wash Buffer I (BD Bioscience), cells permeabilized in Phosflow Perm/Wash Buffer I were stained with anti-phosphatidylserine antibody (4B6) or biotin-conjugated anti-PIP_3_ antibody (Echelon Biosciences Inc.) for 1 hrs at room temperature. After washing twice with Phosflow Perm/Wash Buffer I, cells were incubated with phycoerythrin-conjugated goat anti-mouse IgG antibody (BD Bioscience) to detect 4B6 or phycoerythrin-conjugated avidin (BD Bioscience) to detect biotin-conjugated anti-PIP_3_ antibody. After washing with Phosflow Perm/Wash Buffer I, cells were analyzed using FACScan (BD Bioscience). CellQuest software (BD Bioscience) was used to acquire and quantify the fluorescence signal intensities. To assess the cells' PS synthesis ability, we obtained a ratio of geometric mean fluorescence (GMF) of PS fluorescence in B lymphoblast after exposure to serum free medium for 24 hrs, over GMF of PS after culturing in normal culture media containing 15% FBS. To measure NRG1-stimulated PIP_3_ production, cells were stimulated with either NRG1α (100 ng/ml) or vehicle in a 5% CO_2_ incubator at 37°C. The reaction was terminated at 0, 5, 10, 15 and 30 min by fixing cells with Phosflow Fix Buffer I (BD Bioscience) for 10 min at 37°C. Summation of positive and negative changes in GMF of PIP_3_ fluorescence over baseline during a 30 min observation period was calculated to estimate the extent of NRG1-stimulated PIP_3_ production.

### Overexpression of COMT

Transfection of pAcGFP-N1-COMT (Supplementary Information, Method S1 online) was achieved by the use of Lipofectamine 2000 (Invitrogen) in six-well plates or 4-well chamber slides (Nalge Nunc International, Naperville, IL) according to the manufacturer's recommendation. The cells were transfected at 60% confluency. Transfection efficiencies generally ranged between 70-80% of cells being transfected, as assessed by GFP expression under fluorescence microscope. Optimal transfection consistently resulted in a 4- to 5-fold increase in COMT activity as assessed by enzyme assay (Supplementary Information, Method S1 online) and Western blotting of COMT from cell lysates. Cells were left for 2 days following transfection, before quantification of AKT1 phosphorylation or PS staining.

### PHD-AKT translocation assay

Transfection of pDsRed-ph-AKT or pEYFP-ph-AKT (Supplementary Information, Method S1 online) into SH-SY5Y or B lymphoblasts was achieved using Lipofectamine 2000 (Invitrogen) in poly-L-lysine coated glass 4-well chamber slides (Nalge Nunc International) according to the manufacturer's recommendation. The AKT1 translocation assay was performed one day after transfection. The transfected cells were stimulated with NRG1α (100 ng/ml) for 15 min in an incubator (95% air/5% CO_2_ at 37°C), fixed with 4% paraformaldehyde and examined under a Nikon Eclipse E400 fluorescence microscope (Nikon, Japan) or a LSM 510 confocal laser-scanning microscope (Zeiss, Germany). The transfected cells in which over 70% of PHD-AKT fluorescence was located at the plasma membrane were counted as translocation -positive. Translocation-positive % in stimulated cells/baseline translocation-positive % in unstimulated cells ratio was calculated and used as a translocation index in response to the NRG1 stimulation.

PHD-AKT1 translocation was also examined in COMT-transfected SH-SY5Y cells in order to determine the effects of high COMT enzyme activity on AKT1 translocation ability. For this purpose, the cells were transfected with a combination of two plasmids, either pDsRed-ph-AKT plus pAcGFP-N1-COMT or pDsRed-ph-AKT plus control vector. Then AKT1 the translocation assay was performed 48 hrs after the transfection. The transfected cells were stimulated with NRG1α (25 ng/ml) for 10 min in an incubator (95% air/5% CO at 37 C), fixed with Phosflow Fix Buffer (BD Bioscience) and examined under the fluorescence microscope. In contrast to B lymphoblasts, SH-SY5Y cells indicated distinctive patterns of PHD-AKT1 translocation, which were recognized as multiple spots, clusters or broad membranous. The changes in proportion of cells with these patterns before and after NRG1 treatment were measured and analyzed.

### Migration assay using transwell migration methods

The migration assay was carried out using a transwell chamber with an 8-µm pore size (Costar #3422, Corning, NY). Briefly, cells were suspended at 4×10^5^ cells/ml in serum-free RPMI 1640 with or without 1 mM S-(5′-Adenosyl)-L-methionine chloride (A7007 Sigma-Aldrich, St. Louis, MO), Then, 100 µl of the cell suspension (40,000 cells) were applied to the upper wells of the transwell chamber and incubated in an incubator (95% air/5% CO_2_ at 37°C) for 1 hr. Serum-free RPMI 1640 with or without NRG1 (0.5 ml/well) was then added to the lower wells. After 4 h in an incubator (95% air/5% CO_2_ at 37°C), cells attached to the lower side of the membrane were detached by dissociation buffer (Trevigen, Gaithersburg, MD), lysed with 0.1% Triton X-100 and measured using CyQUANT GR double-stranded DNA detecting reagent (Molecular Probes, Eugene, Oreg). The results were expressed as a chemotaxis index calculated by the following formula: chemotaxis index  =  migration in response to NRG1/migration in response to vehicle control (baseline count). All assays were done in quaduplicate.

### Statistical Analysis

Case-control comparisons for allele frequencies were carried out by chi-square test and Fisher's exact test. The standard measure of linkage disequilibrium (LD), denoted as D' or R^2^, was estimated with Haploview 4.0 in the web http://www.broad.mit.edu/mpg/haploview. A P value of 0.05 was considered significant in tests for Hardy-Weinberg equilibrium. Between groups comparisons were performed by using unpaired *t* tests, or Wald-Wolfowitz runs test, where applicable. The effect of genotype was assessed by ANOVA and followed by Fisher's post-hoc comparison matrices. The parametric relationship between the number of target allele and various phenotypes was assessed using regression analysis. Data were expressed as mean ± SEM.

## Supporting Information

Figure S1 The genomic structure and locations of SNPs in the human AKT1 gene. Numbers in parentheses indicate allele frequencies. Numbers underlined indicate chromosomal positions. The LD map of the AKT1 region in this study (64 Caucasians) was obtained through Haploview v 4.0. Pairwise LD is indicated by the numbers of either D' (left) or r-square (right) and depth of red color.
(0.35 MB TIF)

Figure S2 Effects of COMT Val/Met and AKT1 rs1130233 G/A (G/G = circle, G/A = triangle) genotypes on NRG1-stimulated PIP3 production. Methods to estimate NRG1-stimulated PIP3 production are described in the Materials and Methods section.
(0.03 MB TIF)

Figure S3 Effects of COMT transfection on total PS amount in HEK293 cells. HEK293 cells were transfected with COMT-GFP or with control GFP vector and cultured in media containing 2%FBS. After 48 hrs, cells were analyzed to estimate the total amount of PS by flow cytometry. (A) A two-parameter histogram represents the expression of PS and transfected GFP. (B) An overlay histogram represents both external and internal expression of PS in COMT-transfected cells (filled in black) and control vector-transfected cells (unfilled) in the gated region (squares in the two-parameter histograms). (C) The ratio of the proportion of cells in the upper right quadrant compared to that in the lower right quadrant in COMT-transfected cells was significantly lower than in cells transfected with the control vector (P = 0.0191). (D) PS fluorescence intensity in the gated region (squares in the two-parameters histogram) was significantly decreased in cells transfected with COMT compared with cells transfected with control vector (p = 0.0319).
(0.08 MB TIF)

Figure S4 Effects of COMT transfection, S-adenosylhomocysteine hydrolase (SAHH) transfection and SAM treatment on total PS amount in HEK293 cells. HEK293 cells were double-transfected with COMT-GFP or SAHH-XL5 and their control vectors (empty GFP or empty XL5, respectively) and cultured in media containing 2%FBS. After 48 hrs, cells were treated with 1 mM SAM or vehicle for 60 min and analyzed to estimate the total amount of PS by flow cytometry. A graph indicates the ratio of the proportion of cells in the upper right quadrant (high PS) to the lower right quadrant (low PS) in transfected cells. A higher ratio corresponds to higher total PS. ANOVA showed a significant COMT transfection effect (p = 0.0017) and a SAM treatment effect (p = 0.0192), while there was no effect of SAHH transfection (p = 0.5124) on total PS.
(0.05 MB TIF)

Figure S5 Expression of PSS1, PSS2 and PEMT in B lymphoblasts. Phospholipids biosynthetic pathways. PC, phosphatidylcholine; PE, phosphatidylethanolamine; PEMT, phosphatidylethanolamine N- methyltransferase; PS, phosphatidylserine; PSS, phosphatidylserine synthase. Agarose gel electrophoresis of PCR products following RT-PCR for PSS1, PSS2 and PEMT shows expression of these transcripts in B lymphoblasts. The amplicons from human brain tissue in the first left lane serve as positive controls for these amplifications. RT-PCR. Total RNA was extracted using the SV Total RNA Isolation System (Promega, Madison, WI) and reverse transcription performed to generate the first strand of cDNA using a cDNA synthesis kit (Promega). Synthesized cDNA was then amplified by PCR using following primer sets. The sequences of primers and the annealing temperature for PSS1, PSS2 and PEMT amplifications were: 5′-ATGTGATCACCTGGGAGAGG-3′ (sense) and 5′-CCATTGCACAACAGGATGTC-3′ (antisense) at 55oC, 5′-GGCTCGTCTTCTTCGTGAAC-3′ (sense) and 5′-GATGTAGAAGGGCAGGGACA-3′ (antisense) at 58oC, 5′-AAGACCCGCAAGCTGAGCA-3′ (sense) and 5′-AGTACATGGGGTTGTCCAGGA-3′ (antisense) at 58oC, respectively. The amplification was performed with negative and positive controls using the optimal number of cycles to ensure that the amplification was completed within the exponential range.
(0.10 MB TIF)

Figure S6 Effects of disease and AKT1 rs1130233 on AKT1 transcripts. It should be noted that, although there were no differences in AKT1 protein levels or NRG1-stimulated phosphorylation between lymphoblasts from patients and controls, two-way ANOVA showed significant effects of disease on AKT1 mRNA levels (p = 0.0263), but no effects of AKT1 rs1130233 (P = 0.1514) or no interactions between disease and rs1130233 (p = 0.4275). It is unknown whether or not this elevation reflects compensatory mechanism(s) of unknown defects related to AKT1. * Y-axis, AKT1 mRNA, qPCR results normalized to the geometric mean of three house keeping genes. Real-time quantitative PCR. Real-time RT-PCR reactions for AKT1 mRNA expression were performed using the TaqMan gene expression assay system. First-strand cDNA equivalent to 90 ng of total RNA was prepared in a final volume of 10 µl of Master Mix (Eurogentec, San Diego, CA) with a validated Taqman primer/probe mixture specific to the AKT1 gene (Hs00920503_m1) (Applied Biosystems, Foster City, CA). Real-time RT-PCRs for three housekeeping genes, GUSB, B2M, and ACTB were also performed using TaqMan primer-probes Hs99999908_m1, Hs99999907_m1 and Hs99999903_m1, respectively, to obtain a geometric mean normalization factor (NF3). Real-time RT-PCR reactions were performed in triplicate in a 384-well plate using an ABI Prism 7900 HT sequence detection system. All PCR cycles comprised 2 min polymerase activation at 50°C followed by 10 min denaturation at 95°C and 40 cycles of 95°C for 15 sec and 60°C for 60 sec. Standard curves were established using a pooled cDNA stock from 60 individuals. The cycle threshold (Ct) was determined using SDS 2.0 software (Applied Biosystems). Relative mRNA level of the AKT1 gene in each sample was expressed as a Ct ratio to NF3 (the geometric mean of Ct values from three reference genes) according to the normalization method previously described by Vandesompele et al 45. 45. Vandesompele, J. et al. Accurate normalization of real-time quantitative RT-PCR data by geometric averaging of multiple internal control genes. Genome biology 3, RESEARCH0034 (2002).
(0.04 MB TIF)

Figure S7 A hypothetical mechanisms for COMT-mediated effects on biological functions. The high enzyme activity COMT Val, whose effects can be enhanced by high concentrations of substrates, reduces the SAM pool and increases SAH, which acts as an inhibitor for SAM-dependent methyltransferases. In this manner, PEMT might be affected by COMT activity. A reduction of PEMT activity could change the status of phospholipid synthesis and result in reductions in the PS component of the plasma membrane, which could affect pleckstrin homology domain-mediated binding of protein kinases such as AKT1. Therefore, high COMT could affect not only dopamine or estrogen metabolism, but also other functions mediated by SAM-dependent methyltransferases. Reduced AKT1 responses are expected in individuals with COMT Val than Met homozygotes, as demonstrated in this study. COMT-mediated effects would also be expected to be more robust when there are deficits in essential cofactors such as cholin, B6, B12 and folate and/or when COMT enzyme substrates e.g., high catechols, steroids and related drugs are high.
(0.08 MB TIF)

Method S1 Supplementary Information
(0.10 MB PDF)
