# Supplementary figures and images for: Epistatic and Functional Interactions of Catechol-O-Methyltransferase (COMT) and AKT1 on Neuregulin1-ErbB Signaling in Cell Models
Source: PLoS One. 2010 May 24;5(5):e10789. doi: 10.1371/journal.pone.0010789 (PMC2875391; doi:10.1371/journal.pone.0010789)

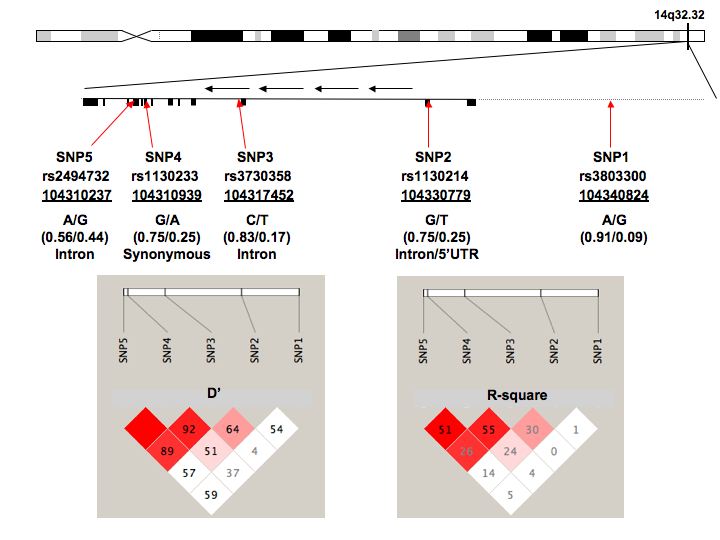

Supplement: Figure S1 — The genomic structure and locations of SNPs in the human AKT1 gene. Numbers in parentheses indicate allele frequencies. Numbers underlined indicate chromosomal positions. The LD map of the AKT1 region in this study (64 Caucasians) was obtained through Haploview v 4.0. Pairwise LD is indicated by the numbers of either D' (left) or r-square (right) and depth of red color. (0.35 MB TIF) [file pone.0010789.s001.tif]

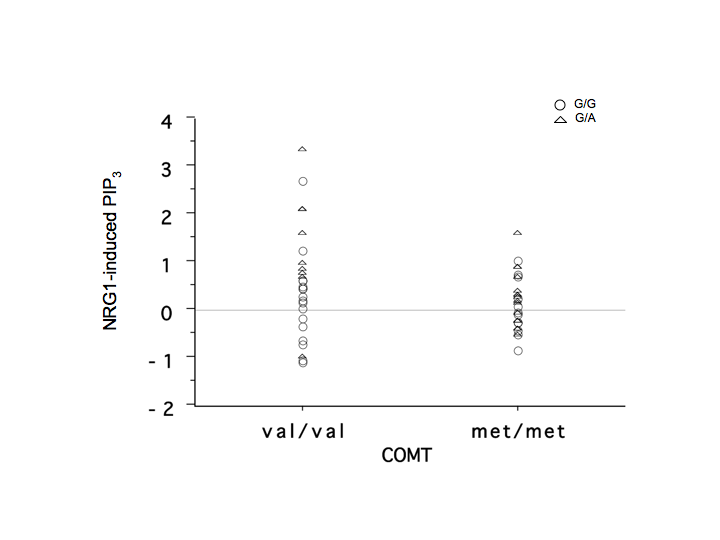

Supplement: Figure S2 — Effects of COMT Val/Met and AKT1 rs1130233 G/A (G/G = circle, G/A = triangle) genotypes on NRG1-stimulated PIP3 production. Methods to estimate NRG1-stimulated PIP3 production are described in the Materials and Methods section. (0.03 MB TIF) [file pone.0010789.s002.tif]

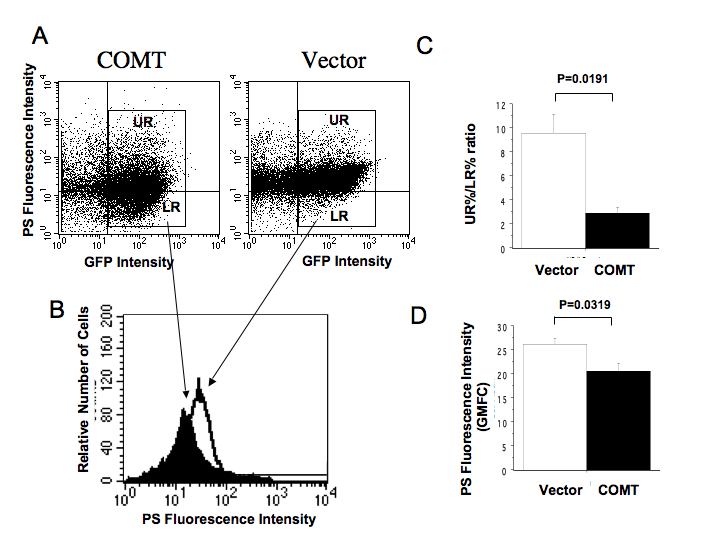

Supplement: Figure S3 — Effects of COMT transfection on total PS amount in HEK293 cells. HEK293 cells were transfected with COMT-GFP or with control GFP vector and cultured in media containing 2%FBS. After 48 hrs, cells were analyzed to estimate the total amount of PS by flow cytometry. (A) A two-parameter histogram represents the expression of PS and transfected GFP. (B) An overlay histogram represents both external and internal expression of PS in COMT-transfected cells (filled in black) and control vector-transfected cells (unfilled) in the gated region (squares in the two-parameter histograms). (C) The ratio of the proportion of cells in the upper right quadrant compared to that in the lower right quadrant in COMT-transfected cells was significantly lower than in cells transfected with the control vector (P = 0.0191). (D) PS fluorescence intensity in the gated region (squares in the two-parameters histogram) was significantly decreased in cells transfected with COMT compared with cells transfected with control vector (p = 0.0319). (0.08 MB TIF) [file pone.0010789.s003.tif]

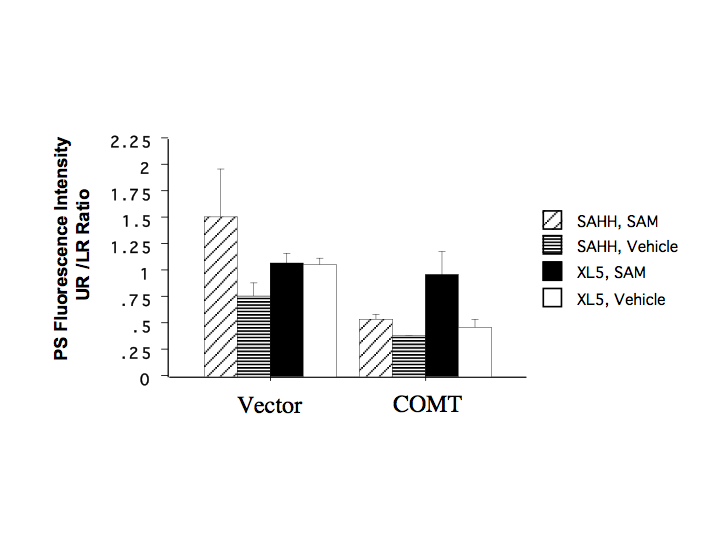

Supplement: Figure S4 — Effects of COMT transfection, S-adenosylhomocysteine hydrolase (SAHH) transfection and SAM treatment on total PS amount in HEK293 cells. HEK293 cells were double-transfected with COMT-GFP or SAHH-XL5 and their control vectors (empty GFP or empty XL5, respectively) and cultured in media containing 2%FBS. After 48 hrs, cells were treated with 1 mM SAM or vehicle for 60 min and analyzed to estimate the total amount of PS by flow cytometry. A graph indicates the ratio of the proportion of cells in the upper right quadrant (high PS) to the lower right quadrant (low PS) in transfected cells. A higher ratio corresponds to higher total PS. ANOVA showed a significant COMT transfection effect (p = 0.0017) and a SAM treatment effect (p = 0.0192), while there was no effect of SAHH transfection (p = 0.5124) on total PS. (0.05 MB TIF) [file pone.0010789.s004.tif]

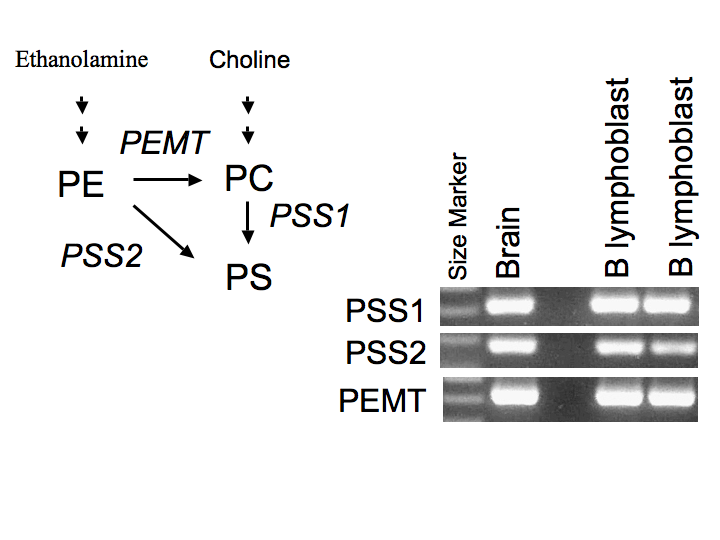

Supplement: Figure S5 — Expression of PSS1, PSS2 and PEMT in B lymphoblasts. Phospholipids biosynthetic pathways. PC, phosphatidylcholine; PE, phosphatidylethanolamine; PEMT, phosphatidylethanolamine N- methyltransferase; PS, phosphatidylserine; PSS, phosphatidylserine synthase. Agarose gel electrophoresis of PCR products following RT-PCR for PSS1, PSS2 and PEMT shows expression of these transcripts in B lymphoblasts. The amplicons from human brain tissue in the first left lane serve as positive controls for these amplifications. RT-PCR. Total RNA was extracted using the SV Total RNA Isolation System (Promega, Madison, WI) and reverse transcription performed to generate the first strand of cDNA using a cDNA synthesis kit (Promega). Synthesized cDNA was then amplified by PCR using following primer sets. The sequences of primers and the annealing temperature for PSS1, PSS2 and PEMT amplifications were: 5′-ATGTGATCACCTGGGAGAGG-3′ (sense) and 5′-CCATTGCACAACAGGATGTC-3′ (antisense) at 55oC, 5′-GGCTCGTCTTCTTCGTGAAC-3′ (sense) and 5′-GATGTAGAAGGGCAGGGACA-3′ (antisense) at 58oC, 5′-AAGACCCGCAAGCTGAGCA-3′ (sense) and 5′-AGTACATGGGGTTGTCCAGGA-3′ (antisense) at 58oC, respectively. The amplification was performed with negative and positive controls using the optimal number of cycles to ensure that the amplification was completed within the exponential range. (0.10 MB TIF) [file pone.0010789.s005.tif]

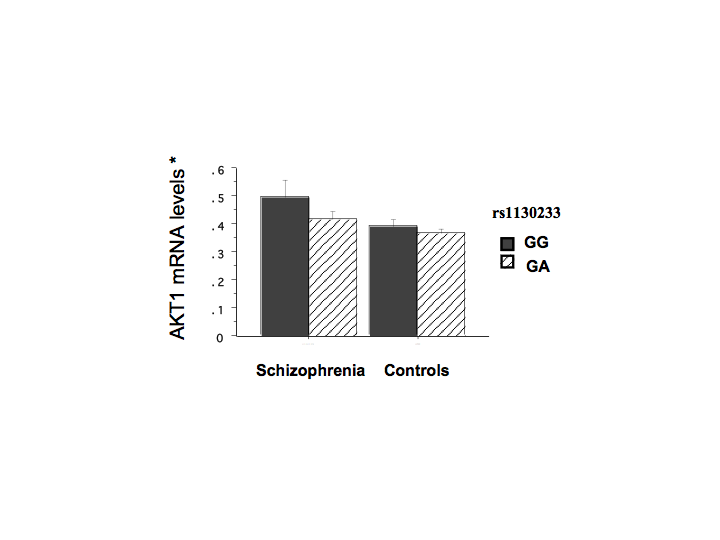

Supplement: Figure S6 — Effects of disease and AKT1 rs1130233 on AKT1 transcripts. It should be noted that, although there were no differences in AKT1 protein levels or NRG1-stimulated phosphorylation between lymphoblasts from patients and controls, two-way ANOVA showed significant effects of disease on AKT1 mRNA levels (p = 0.0263), but no effects of AKT1 rs1130233 (P = 0.1514) or no interactions between disease and rs1130233 (p = 0.4275). It is unknown whether or not this elevation reflects compensatory mechanism(s) of unknown defects related to AKT1. * Y-axis, AKT1 mRNA, qPCR results normalized to the geometric mean of three house keeping genes. Real-time quantitative PCR. Real-time RT-PCR reactions for AKT1 mRNA expression were performed using the TaqMan gene expression assay system. First-strand cDNA equivalent to 90 ng of total RNA was prepared in a final volume of 10 µl of Master Mix (Eurogentec, San Diego, CA) with a validated Taqman primer/probe mixture specific to the AKT1 gene (Hs00920503_m1) (Applied Biosystems, Foster City, CA). Real-time RT-PCRs for three housekeeping genes, GUSB, B2M, and ACTB were also performed using TaqMan primer-probes Hs99999908_m1, Hs99999907_m1 and Hs99999903_m1, respectively, to obtain a geometric mean normalization factor (NF3). Real-time RT-PCR reactions were performed in triplicate in a 384-well plate using an ABI Prism 7900 HT sequence detection system. All PCR cycles comprised 2 min polymerase activation at 50°C followed by 10 min denaturation at 95°C and 40 cycles of 95°C for 15 sec and 60°C for 60 sec. Standard curves were established using a pooled cDNA stock from 60 individuals. The cycle threshold (Ct) was determined using SDS 2.0 software (Applied Biosystems). Relative mRNA level of the AKT1 gene in each sample was expressed as a Ct ratio to NF3 (the geometric mean of Ct values from three reference genes) according to the normalization method previously described by Vandesompele et al 45. 45. Vandesompele, J. et al. Accurate normalization [file pone.0010789.s006.tif]

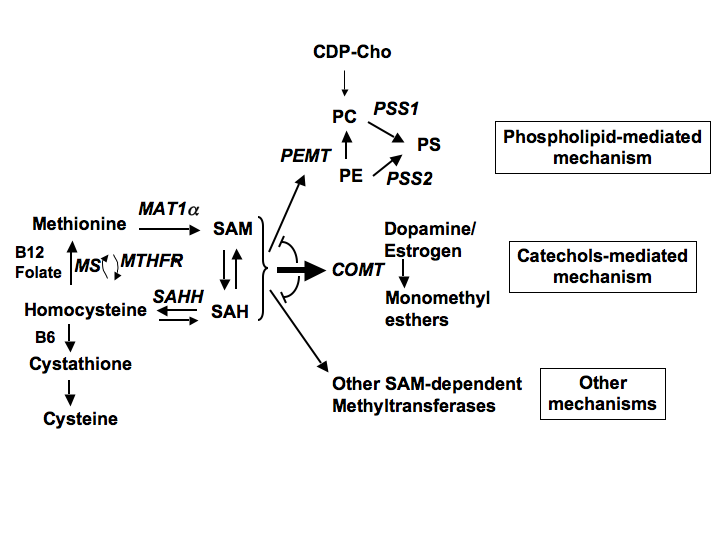

Supplement: Figure S7 — A hypothetical mechanisms for COMT-mediated effects on biological functions. The high enzyme activity COMT Val, whose effects can be enhanced by high concentrations of substrates, reduces the SAM pool and increases SAH, which acts as an inhibitor for SAM-dependent methyltransferases. In this manner, PEMT might be affected by COMT activity. A reduction of PEMT activity could change the status of phospholipid synthesis and result in reductions in the PS component of the plasma membrane, which could affect pleckstrin homology domain-mediated binding of protein kinases such as AKT1. Therefore, high COMT could affect not only dopamine or estrogen metabolism, but also other functions mediated by SAM-dependent methyltransferases. Reduced AKT1 responses are expected in individuals with COMT Val than Met homozygotes, as demonstrated in this study. COMT-mediated effects would also be expected to be more robust when there are deficits in essential cofactors such as cholin, B6, B12 and folate and/or when COMT enzyme substrates e.g., high catechols, steroids and related drugs are high. (0.08 MB TIF) [file pone.0010789.s007.tif]
