# Supplementary material for: Epistatic and Functional Interactions of Catechol-O-Methyltransferase (COMT) and AKT1 on Neuregulin1-ErbB Signaling in Cell Models
Source: PLoS One. 2010 May 24;5(5):e10789. doi: 10.1371/journal.pone.0010789 (PMC2875391; doi:10.1371/journal.pone.0010789)
Supplement: Method S1 — Supplementary Information (0.10 MB PDF) [file pone.0010789.s008.pdf]

Supplementary Information, Method

Construction of pAcGFP-N1-COMT

An upstream primer, 5'-GGCAAGCTTTGAAGATGCCGGAGGCCCCGCCT-3', engineered with a (Hind III) restriction site (underlined), and a downstream primer, 5'-GGCGGATCCCAGGGCCCTGCTTCGCTGCCT-3', engineered with a (Bam H1) restriction site (underlined), were used to PCR amplify the full-length MB human COMT open reading frame from human COMT-Val158 cDNA (Genbank ID: BC011935) plasmid (pOTB7-COMT-Val) (American Type Culture Collection). The amplicon was digested with HindIII and BamH1and subcloned into the plasmid pAcGFP-N1 (BD Bioscience).

Construction of PHD-AKT.

A Discosoma genus red protein-tagged expression vector for PHD of human AKT1 gene was constructed using RT-PCR following by subcloning. Primers 5'-CGGGCACCATGAGCGACGT-3' and 5'-CCGTCAGCCACAGTCTGCATG-3' were used to first amplify the PH-domain of AKT1 gene from HEK293 cell cDNA. The purified PCR product was re-amplified using the primers 5'-CGGGATCCATGAGCGACGTGGCTATTGTGAA-3' and 5'-CTAGAGGCCGTCAGCCACAGTCTGGATG-3'. To generate the insert, the PCR product was treated with polynucleotide kinase, digested with BamHI and gel-purified. The purified insert was then subcloned into pDsRed Mono C1 or pEYFP from Clontech (Heidelberg, Germany) at blunted XbaI and BamHI sites, and the sequence was verified by sequencing.

Modified COMT activity assay.

The COMT enzyme activity assay was based on the organic solvent extraction method that separates the radioactive product, the methylated catechol, and the free radioactive co-enzyme, ^{3}H-S-adenosyl-methionine [46]. 50 µg of protein was transferred to a fresh microcentrifuge tube from each sample. 500 µL of the substrate mixture containing 10 mM Tris, pH 7.4, 1 mM MgCl2, 1.5 µCi of ^{3}H-adenosyl-S-methionine, 10 µM of catechol and 1 µM of DTT was added to each tube. The tubes were then incubated at 37 °C for 20 min. The reactions were immediately terminated by adding 500 µl of 1M HCl. The radioisotope-labeled catechol products from the reactions were extracted and determined by mixing the reaction mixture with 10 ml scintillation fluid (Flow I, Molecular Diagnosis) and measuring the radioactivity of the mixture in a scintillation counter. Relative COMT enzyme activity was presented as DPM per mg total protein (or percentage). To establish a baseline control for non-specific reactions that do not depend on COMT, 100µM COMT inhibitor, tolcapone, was added to a tube containing 50 µg of the protein sample. The high concentration of a potent COMT inhibitor blocked the specific reaction catalyzed by COMT, and the radioactivity from this reaction served as a baseline. Relative COMT enzyme activity in the COMT transfected cells and their vector transfected controls were measured and presented as radioactivity of the ^{3}H-labeled methylated product in DPM per mg of total protein (or percentage).

46. Zurcher, G. & Da Prada, M. Rapid and sensitive single-step radiochemical assay for catechol-O-methyltransferase. *Journal of neurochemistry* 38, 191-195 (1982)
